# Impact of Laparoscopic Partial Nephrectomy and Open Partial Nephrectomy on Outcomes of Clear Cell Renal Cell Carcinoma

**DOI:** 10.3389/fsurg.2021.681835

**Published:** 2021-07-29

**Authors:** Feng Yu, Qian Xu, Xian-Gen Liu

**Affiliations:** Department of Urology, Nantong Third People's Hospital, Nantong, China

**Keywords:** kidney cancer, complex kidney cancer, laparoscope, partial nephrectomy, serum creatinine

## Abstract

**Objective:** To analyze the impact of laparoscopic partial nephrectomy (LPN) and open partial nephrectomy (OPN) on outcomes of complex clear cell renal cell carcinoma (ccRCC).

**Methods:** A total of 132 high-complex ccRCC patients with a Radius Exophytic Nearness Anterior Location (R.E.N.A.L) score ≥7 enrolled in our hospital between January 2018 and June 2020 were matched and assigned to an LPN group (given LPN treatment) and an OPN group (given OPN treatment), with 66 cases in each group. Two weeks and 3 months after the operation, the renal indexes, inflammatory factors, basic perioperative conditions, and incidence of complications were compared.

**Results:** Two weeks after the operation, the levels of SCr and CysC were elevated, with higher levels observed in the LPN group (all *P* < 0.05), and the eGFR levels were reduced, with a lower result in the LPN group. Three months after the operation, the two groups observed decreased levels of SCr and CysC, and an increased level of eGFR; moreover, the decreased SCr and CysC levels were still higher, and the increased eGFR was lower than those before the operation (*P* < 0.05). The levels of CRP and TNF-α in the two groups increased after the operation, with a lower outcome in the LPN group (*P* < 0.05). Moreover, the LPN group had less intraoperative blood loss and shorter postoperative length of hospital stay but longer blocking time compared to the OPN group (*P* < 0.05). Patients in the LPN group were recorded with a lower complication incidence compared with the OPN group (3.03 vs. 15.15%, *P* < 0.05).

**Conclusion:** Both LPN and OPN enjoy significant efficacy in the treatment of complex ccRCC and effectively protect renal function. Moreover, LPN is a more acceptable option for complex ccRCC due to its numerous benefits in postoperative stress response, complications, recovery. which is worthy of promotion with safety and feasibility.

## Introduction

Kidney cancer is a common urinary system cancer, with prevalence second to bladder cancer (1). Renal cancer is a malignant tumor caused by the cancerization of epithelial cells in different parts of the urinary tubules in the renal parenchyma. Its etiology is related to obesity, smoking, high blood pressure, long-term use of hormones, antipyretic and analgesic drugs, and other factors (2). Renal parenchymal carcinoma is an adenocarcinoma derived from renal tubular epithelial cells, 85% of which are clear cell carcinoma, and some are granular cell carcinoma and mixed cell carcinoma, with common manifestations of hemorrhage, necrosis, cystic transformation, and calcification. Born in the kidney parenchyma, it infiltrates, compresses, and destroys the renal pelvis and calyces after growing up, develops outside the renal capsule, forms hemangioma thrombus, or metastasizes to lymph nodes and other organs. As imaging technology advances, the diagnosis accuracy of early kidney cancers has been witnessing an upward trend in recent years. Studies have found that kidney cancers are not sensitive to radiation with several drug resistances, and targeted therapy and immunotherapy harbor certain restrictions as well (3); thus surgical resection remains to be the mainstay.

Nephron sparing surgery (NSS) is currently considered to be an optimal technique in clinical practice (4). It not only yields similar efficacy with radical nephrectomy (RN) but also preserves nephrons to provide patients with a satisfactory postoperative quality of life (5). As clinical research deepens, laparoscopic partial nephrectomy (LPN), robot-assisted partial nephrectomy (RAPN), open partial nephrectomy (OPN), and the indication scope have also been developed (6). OPN and LPN have been widely used in clinical practice in China due to their lower requirements for surgical equipment, and their similar efficacy has also been proven (7). However, in terms of the removal of nephrons in the treatment of complex clear cell renal cell carcinoma (ccRCC) which are deeply surrounded by renal parenchyma, located near the midline of the renal coronal plane, and relatively close to the kidney collecting system with complex anatomical structures, their efficacy is critically challenged and remains controversial. OPN is criticized for its large incision, strict requirements for postoperative analgesia, obvious scars, and long recovery time (8). With the continuous advancement of minimally invasive technology, the internal suture techniques have been improved, the intraoperative hemostatic materials and LPN have been increasingly used in surgery to preserve kidney function. In view of this, we compared the effects of LPN and OPN in the treatment of complex ccRCC in the present study.

## Materials and Methods

### General Materials

This study enrolled 132 patients with complex ccRCC treated in our hospital between 2018 and June 2020. Inclusion criteria: (1) Patients whose imaging examinations, clinical symptoms, histology tests, and laboratory tumor markers met with the clinical diagnostic criteria of ccRCC; (2) Patients with 7 points or more in Radius Exophytic Nearness Anterior Location (R.E.N.A.L.), Preoperative Aspects and Dimensions Used for Anatomic (PADUA) > 10 points; (3) Patients met the treatment indications of OPN and LPN; (4) Patients over 18 years; (5) Patients who voluntarily signed informed consent letter. Exclusion criteria: (1) Patients with distant metastasis; (2) Patients with ≥4 cm tumor diameter; (3) Patients with poor kidney functions; (4) Patients with an abnormal renal anatomical structure such as horseshoe kidney, solitary kidney; (5) Patients with contraindications related to surgery or anesthesia; (6) Patients who lost contact or died during the follow-up. Logistic regression was used to score propensity matching. The basic conditions of the two groups of patients included gender, age, R.E.N.A.L. classification, and PADUA scoring system. The two groups were used as 1/0 binary processing indicators, gender, age, and R.E.N.A.L. classification was used as co-variables, and the propensity score matching standard (caliper value) was set to 0.02. Logistic regression was used to score the propensity matching, with a matching ratio of 1:1, and the cases with similar scores were matched, with an LPN group (*n* = 66) and an OPN group (*n* = 66). There were 36 males and 30 females in the LPN group whose average age was (52.16 ± 8.43) years old, with the average tumor diameter of (2.76 ± 0.81) cm, a mean BMI of (24.6 ± 3.1) kg/m2, 31 cases of American Society of Anesthesiologists (ASA) stage 3, 35 cases of stage 4, 40 cases of hypertension, and 39 cases of diabetes; There were 32 cases with left kidney disease and 34 cases with right kidney disease, 45 cases with moderately complex ccRCC and 21 cases with severely complex ccRCC. There were 37 males and 29 females in the OPN group whose average age was (53.46 ± 8.51) years old, with the average tumor diameter of (2.80 ± 0.76) cm, a mean BMI of (25.0 ± 2.9) kg/m2, 30 cases of ASA stage 3, 36 cases of stage 4, 41 cases of hypertension, and 40 cases of diabetes; There were 33 cases with left kidney disease and 33 cases with right kidney disease, 46 cases with moderately complex ccRCC and 20 cases with severely complex ccRCC. The two groups obtained similar general information (*P* > 0.05). The research followed ethical standards, was approved by the ethics committee (ethics certificate number: 2017-11-25), and followed the biosafety and institutional safety procedures.

### Methods

Inspection method: flat scan plus enhancement. The non-ionic contrast agent for enhancement was injected through the cubital vein with a high-pressure syringe at a flow rate of 2~3 m/s. The arterial phase scan was started at 40 s, the parenchymal scan was performed at 150 s, and the excretion phase scan was performed 5 min later.

Single-slice spiral CT scanner, matrix 512 × 512, slice thickness and interval were 10 mm conventionally, and 3~5 mm thin-slice scanning for small lesions; 4-slice spiral CT scanner, matrix 512 × 512, layer thickness and interval were 5 mm, and 1~3 mm thin-slice reconstruction was performed when necessary. All cases used multi-phase scanning, that is, single-slice spiral CT machine plain scan, enhanced, and delayed scan; multi-slice spiral CT machine plain scan, arterial phase, parenchymal phase, and delayed scan.

All the patients in this study underwent routine gastrointestinal decompression and general anesthesia for tracheal intubation and were placed in a lateral position with the waist moderately underlay. In the OPN group, we made a 15–20 cm oblique incision under the 11th intercostal or 12th rib and pushed the retroperitoneal approach inward to separate the subcutaneous tissues, internal oblique muscle, external oblique muscle, low back muscle, transverse abdominis, and the prerenal fascia. Following the actual condition, we exposed the kidney and opened other tissues, the adjacent adipose capsule, and renal fascia, and clamped the renal pedicle in the same way for coagulation treatment. Next, we excised with surgical scissors along the 0.5–1.0 cm boundary between the tumor and the normal renal parenchyma. After confirming the resection completion through pathological examination, we sutured it in the same way as to restore blood flow. Subsequently, after the confirmation of non-invasive surface bleeding, we placed a drainage tube and sutured the abdomen.

During the LPN, we made a 2 cm incision at the lower edge of the 12th rib on the posterior axillary line. Next, we separated the muscle and lower back fascia with hemostatic forceps and locate the retroperitoneal cavity. Then we placed a balloon dilator under the guidance of iodophor to infuse 500–700 ml of air to create a posterior abdominal cavity and then held it for 5 min while compressing the bleeding. Subsequently, we made a 2 cm incision at the mid-axillary iliac crest line, punctured below the front axillary line, and placed the corresponding trocar and endoscope. Afterward, we established a 12–15 mmHg of artificial pneumoperitoneum to explore the abdominal environment and identify the psoas major and other landmark structures. Then we cut the extraperitoneal fat with an ultrasonic knife, released the kidney outside, and exposed the renal artery. And we separated the adipose capsule on the surface of the kidney to expose the tumor as much as possible, and carefully identified the boundary direction different from the normal kidney parenchyma. After separating the prerenal fascia and confirming the renal tumor, we freed the back of the kidney to the renal pedicle, blocked the renal pedicle artery with arterial blocking forceps, and then we injected ice water to cool the perinephric temperature. In the end, we cauterized it at 0.5–1.0 cm outside the boundary with the help of an electrocoagulation hook. If it was diagnosed as cancer through pathological puncture before the operation, we removed it together with the renal fascia and placed it in a specimen bag. Next, we took 2–4 specimens outside the edge to rapidly frozen them and conduct the pathological examination. After confirming no remaining tumor, we sutured the vascular stump with absorbable sutures (4–0), the kidney wound with absorbable sutures (3–0) “sandwich method,” and then loosened the blocking forceps. Upon confirming no active bleeding, we retained the drainage tube and sutured the external incision. All patients regularly conducted the examination of renal function after the operation, and we checked the damage or recovery of their kidney and surrounding organs with CT or B-ultrasound.

### Observation Indicators

Venous blood samples before operation and within 2 weeks and 3 months after the operation were collected, coagulated, and centrifuged; the supernatant was then extracted and transferred into a tube. Then one part of the supernatant was used with the automatic biochemical analyzer and its supporting reagents to detect cystatin C (CysC), serum creatinine (SCr), and estimated glomerular filtration rate (eGFR). The rest was determined by enzyme-linked immunosorbent assay (ELISA) with C-reactive protein (CRP), tumor necrosis factor-α (TNF-α) corresponding kits. In the end, we statistically analyzed the basic conditions of patients during the perioperative period, such as intraoperative blood loss, blocking time, postoperative hospital stay, and the incidence of complications within 2 weeks.

### Statistical Methods

SPSS 23.0 was applied to analyze the data and Graphpad prism 8.0 was applied to plot graphs in this study. Count data were expressed as a percentage, using the χ2 test, and measurement data were expressed as (*x*±s), using the *t*-test. Repeated measurement data were conducted by analysis of variance. Significance was determined as a *P* < 0.05. Perform propensity score matching was used to obtain two matched groups.

## Results

### Comparison of SCr Levels

The SCr levels of the two groups before the operation were similar (*P* > 0.05). Within 2 weeks after the operation, SCr levels witnessed a trend of increase, with a higher result in the LPN group (*P* < 0.05). Three months after the operation, the levels of the two groups decreased but were still higher than those before the operation (*P* < 0.05), and the LPN group still yielded a higher level (*P* > 0.05). See Table 1.

**Table 1:** Comparison of SCr level (*x*±s, μmol/L).

**Group**	***n***	**Before operation**	**Two weeks after operation**	**Three months after operation**
LPN group	66	72.16 ± 11.43	127.48 ± 20.19	79.86 ± 12.14
OPN group	66	72.93 ± 11.29	107.46 ± 18.42	77.85 ± 11.96
*F* group			361.622	
*P* group			<0.001	
*F* time			23.034	
*P* time			<0.001	
*F* correlation			19.472	
*P* correlation			<0.001	

### Comparison of Cys-C Levels

No statistical difference was found in the Cys-C levels between the two groups before the operation (*P* > 0.05). Similar to SCr, the changes in the levels of Cys-C observed an increase within 2 weeks after the operation (*P* < 0.05) and then a downturn 3 months after the operation (*P* < 0.05), with constantly higher levels than before the operation (*P* < 0.05) and a lower result in the LPN group (*P* > 0.05). See Table 2.

**Table 2:** Comparison of Cys-C level (*x*±s, mg/L).

**Group**	***n***	**Before operation**	**Two weeks after operation**	**Three months after operation**
LPN group	66	1.58 ± 0.29	2.84 ± 0.72	1.89 ± 0.24
OPN group	66	1.54 ± 0.31	2.41 ± 0.65	1.76 ± 0.18
*F* group			201.034	
*P* group			<0.001	
*F* time			19.623	
*P* time			<0.001	
*F* correlation			6.817	
*P* correlation			<0.001	

### Comparison of eGFR Levels

The two groups showed no great disparity in the eGFR levels before the operation (*P* > 0.05). The eGFR levels saw a slump 2 weeks after the operation (*P* < 0.05), and then up-regulated 3 months after the operation (*P* < 0.05). Moreover, the eGFR levels were constantly lower than before (*P* < 0.05) and the LPN group yielded a lower outcome than the OPN group (*P* > 0.05). See Table 3.

**Table 3:** Comparison of eGFR levels [*x*±s, ml/(min·1. 73 m^2^)].

**Group**	***n***	**Before operation**	**Two weeks after operation**	**Three months after operation**
LPN group	66	75.12 ± 10.24	31.18 ± 7.48	63.48 ± 9.47
OPN group	66	75.86 ± 10.36	38.94 ± 7.59	66.44 ± 9.57
*F* group			687.214	
*P* group			<0.001	
*F* time			17.102	
*P* time			<0.001	
*F* correlation			5.028	
*P* correlation			0.007	

### Comparison of Inflammatory Factors

**Figure 1:**
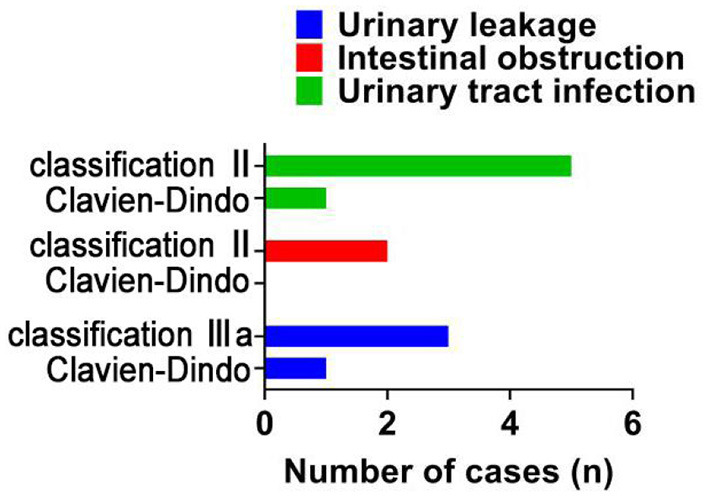
Comparison of incidence of complications.

No statistical difference was identified in the CRP and TNF-α levels in the two groups before the operation (*P* > 0.05). After the operation, there was an increase in the CRP and TNF-α levels of the two groups, with lower levels recorded in the LPN group (*P* < 0.05). See Table 4 and Figure 1.

**Table 4:** Comparison of CRP and TNF-α levels (*x*±s).

**Group**	***n***	**CRP (mg /L)**		**TNF-α** **(ng /L)**	
		**Before operation**	**After operation**	**Before operation**	**After operation**
LPN group	66	1.26 ± 0.29	22.81 ± 3.46^*^	2.89 ± 0.86	23.74 ± 4.88^*^
OPN group	66	1.31 ± 0.27	27.18 ± 3.49^*^	2.84 ± 0.91	28.43 ± 4.41^*^
t		1.025	7.224	0.324	5.793
*P*		0.307	<0.001	0.746	<0.001

* *Means the comparison with before operation (P < 0.05)*.

### Comparison of Perioperative Indicators

The LPN group had less intraoperative blood loss and spent a shorter postoperative hospital stay compared with the OPN group, but longer blocking time (*P* < 0.05). See Table 5.

**Table 5:** Comparison of perioperative indicators (*x*±s).

**Group**	***n***	**Intraoperative blood loss (mL)**	**Blocking time (min)**	**Postoperative hospital stays (d)**
LPN group	66	152.48 ± 20.16	28.15 ± 6.86	10.74 ± 2.15
OPN group	66	201.75 ± 23.95	21.27 ± 4.29	14.46 ± 3.87
*t*		12.792	6.908	6.826
*P*		<0.001	<0.001	<0.001

### Comparison of the Incidence of Complications

**Figure 2:**
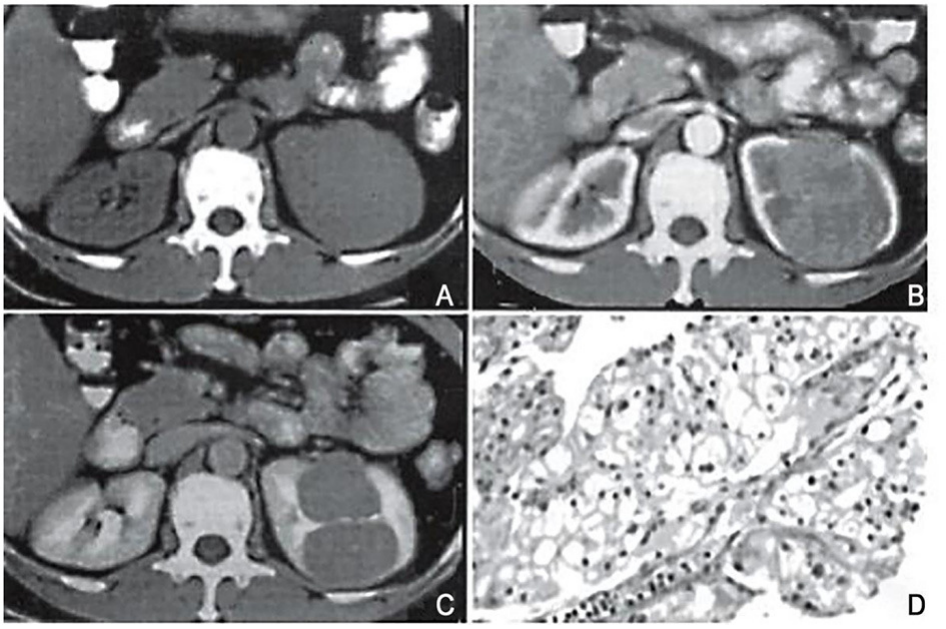
Case 1, female, 51 years old. CT classification: limited convex type. **(A)** Flat scan: an isobaric soft tissue mass in the upper part of the left kidney is partially protruding out of the contour of the kidney, with a smooth edge. The density of the lesion is uniform, and the boundary with the kidney tissue is unclear. **(B)** Arterial phase: the tumor shows obvious uniform enhancement, with clear borders and lobules. The lesion is locally convex but the surface is smooth. **(C)** Delayed scan: the tumor density is reduced, but the borders are clear, and a pseudo-capsule is formed around it. Surgery pathological biopsy (HEx200). The tumor tissue is arranged in a papillary shape. The image is a longitudinal section of a nipple, with a longitudinal section of a blood vessel visible in the center. Cancer cells are round-shaped, with transparent cytoplasm, and nuclei in the center or offset.

There were 3 cases of Clavien-Dindo classification IIIa, 2 cases of Clavien-Dindo classification II, and 5 cases of Clavien-Dindo classification II in the OPN group. There was 1 case of Clavien-Dindo classification IIIa and 1 case of Clavien-Dindo classification II in the LPN group. The LPN group had a lower incidence of complications compared with the OPN group [3.03% (2/66) vs. 15.15% (10/66), *P* < 0.05]. See Figure 2. No cases were converted to nephrectomy.

## Discussion

The location and size of the tumor directly determine the proportion of retained nephrons and the complexity of the NSS design (9). At present, the 7 points or more in R.E.N.A.L are commonly defined as complex ccRCC in clinical practice in China (10). Although the scoring system provides certain guidance and reference for the surgical plan and the prediction of postoperative renal function, no unified standards in clinical practice have yet been achieved (11). Therefore, the feasibility of the current options remains unknown.

Obstruction of the renal pedicle, an inevitable method in NSS, greatly reduces intraoperative blood loss and obtains a clear surgical vision. However, the kidney is in an ischemic state after obstruction, and if the blocking time cannot be controlled, it will lead to postoperative renal damage (12). Currently, laparoscopic surgery has been applied in clinical practice, but LPN requires strict operation skills of the surgeons such as removing tumors, repairing renal pelvis and renal calyx, renal hemostasis, suture techniques in a narrow space under a laparoscope (13). As a result, OPN is more preferred for the treatment of complex ccRCC due to its wide surgical vision and widespread indications (14). The results of this study showed that the LPN group had a significantly longer blocking time compared with the OPN group. Within 2 weeks after the operation, patients were recorded with great damage in the renal function in the RLPN group, but there was no significant difference in the comparison of renal function between groups at 3 months after the operation. Although LPN has more difficulty in operation, longer blocking time, and obvious postoperative early damage in renal function, it still plays a role in the preservation of renal function similar to OPN after a period of recovery, which indicates its feasibility for kidney cancers treatment. In this study, it is necessary to completely free 1 cm renal artery during the obstruction to facilitate clamping, to avoid clamping tissues adjacent to the renal pedicle. Otherwise, it increased bleeding due to incomplete obstruction. Some scholars have proposed (15) that CT angiography or renal color Doppler ultrasound can be used to confirm renal artery branches, which paves the way for the simplified operation of the complete intraoperative blockade, and two artery-blocking forceps can be applied when necessary. OPN in the treatment of various kidney cancers has been widely recognized in clinical practice, but it inevitably results in a long incision and large anatomical area (16). OPN has common characteristics such as postoperative pain, deep surgical wounds, obvious scars after healing. Therefore, the postoperative physiological function of patients is challenged (17).

TNF-α, a pro-inflammatory cytokine with a wide range of biological effects, mediates acute-phase proteins to induce inflammation and excessive immune responses (18). CRP, a non-specific acute-phase protein synthesized by the liver, can promote the incidence and development of systemic or local inflammation (19). The authors found that the increase in inflammatory factors in the LPN group was lower than the OPN group after the operation, indicating that LPN reduced the incision area and the anatomy scope to avoid interference with other organs, and prevent bodies from more stress and trauma, which may boost the recovery of patients after operation.

CysC can be used as a sensitive marker to reflect early renal impairment. The routine renal function test fails to detect the slightly damaged structure and function of the kidney or the damage at the early stage with high sensitivity, and the routine examination of urine protein is also negative at this stage. When BUN and Scr are not yet elevated, kidney damage is already present, which keeps the early diagnosis at bay. Moreover, in the early stage of renal damage in hypertension, no obvious clinical symptoms can be found in the patients, which further underlines the importance of early diagnosis. CysC is therefore considered to be the first choice for evaluating early glomerular filtration function and an early indicator of the severity of end-organ damage in hypertensive patients. CysC as an evaluation index of early renal damage in elderly hypertensive patients can reap huge fruits in the early diagnosis and treatment of renal damage caused by hypertension, help prevent or delay the progression of the disease, which has important clinical significance, and application value. In addition, due to the strong reserve capacity and the compensatory function of the kidney, when the early renal function is impaired, the blood concentration of Scr shows no evident changes, which confirms its impotence of being an indicator of early renal damage.

Creatinine is the end product of creatine and creatine phosphate metabolism. It can be filtered freely through the glomerulus but is rarely absorbed in the renal tubules. Creatinine or the creatinine clearance rate calculated by creatinine can reflect the glomerular filtration rate (GFR), which is used as an index to evaluate renal function. However, there are two types of creatinine in the blood, endogenous, and exogenous. Endogenous creatinine is a product of muscle metabolism in the body, so it is affected by factors other than kidney function such as age, gender, race, and muscle volume. Exogenous creatinine is the product of ingested meat food after metabolism in the body, so it is affected by the amount of meat food intake.

Studies have reported (20) that the stress of tumor resection surgery releases a large number of glucocorticoids, thereby leading to an imbalance of immune function. To a certain extent, it increases pathogen infection and susceptibility and risk of recurrence, with a somber prognosis. We found that the LPN group had less intraoperative blood loss, shorter postoperative hospital stay, and a lower complication rate in comparison with the OPN group. It suggests the better safety of LPN surgery and rapid recovery of the gastrointestinal function and other physiological functions of patients after the operation. The following may explain the main results. First, we plan to reduce the bleeding to ensure a clear surgical vision, avoid damaging other tissues or long-term drainage during operation. Furthermore, we reduce complications caused by bed rest and hospitalization and create conditions for rapid recovery of patients. Nevertheless, we have little experience in renal parenchymal wound tissue, and sutures and knots may result in tissue avulsion, especially when it sutures together with capsules during laparoscopic surgery. Moreover, we carefully control the tightening of the sutures and check the closure of the renal collecting system in order to further reduce complications such as postoperative bleeding, urine leakage, urinary system infection. The limitation of the study is that the follow-up time was shorter than 3 months, the complex location and characteristics of the tumor were not clearly provided, and the factors of the patients' secondary diseases, such as diabetes, hypertension, and risk factors of postoperative renal function limitation, were not further explored, which results in the deficiency of possible standardization; in addition, this study did not conduct predictions based on R.E.N.A.L. and PADUA in terms of complications and conversion to nephrectomy.

In summary, we conclude that LPN is an acceptable option for complex ccRCC due to its numerous benefits in postoperative stress response, complications, recovery.

## Data Availability Statement

The original contributions presented in the study are included in the article/supplementary materials, further inquiries can be directed to the corresponding author.

## Author Contributions

All authors listed have made a substantial, direct and intellectual contribution to the work, and approved it for publication.

## Conflict of Interest

The authors declare that the research was conducted in the absence of any commercial or financial relationships that could be construed as a potential conflict of interest.

## Publisher's Note

All claims expressed in this article are solely those of the authors and do not necessarily represent those of their affiliated organizations, or those of the publisher, the editors and the reviewers. Any product that may be evaluated in this article, or claim that may be made by its manufacturer, is not guaranteed or endorsed by the publisher.

## References

[1] Taylor AS Spratt DE Dhanasekaran SM Mehra R. Contemporary renal tumor categorization with biomarker and translational updates: a practical review. Arch Pathol Lab Med. (2019) 143:1477–91. 10.5858/arpa.2019-0442-RA 31765248

[2] Herrera-Caceres JO Finelli A Jewett MAS. Renal tumor biopsy: indicators, technique, safety, accuracy results, and impact on treatment decision management. World J Urol. (2019) 37:437–43. 10.1007/s00345-018-2373-9 30022406

[3] Yamagami T Yoshimatsu R Kajiwara K Yamanishi T Minamiguchi H Karashima T Inoue K. Protection from injury of organs adjacent to a renal tumor during percutaneous cryoablation. Int J Urol. (2019) 26:785–90. 10.1111/iju.14013 31094038

[4] Fetahu A Çuni X Haxhiu I Çuni L Manxhuka S Shahini L. Open nephron sparing surgery for T1a renal tumors: clinical experience in an emerging Country. Gulf J Oncolog. (2019) 1:60–5. 31591992

[5] Carcinoma de células renales en la enfermedad de von Hippel-Lindau; cirugía conservadora de nefronas [Renal cell carcinoma in von Hippel-Lindau disease. Nephron sparing surgery]. Arch Esp Urol. (2018) 71:757–64. 30403378

[6] Jilg CA Neumann HP Gläsker S Schäfer O Leiber C Ardelt PU. Nephron sparing surgery in von Hippel-Lindau associated renal cell carcinoma; clinicopathological long-term follow-up. Fam Cancer. (2012) 11:387–94. 10.1007/s10689-012-9525-7 22426863

[7] Meyer A Woldu SL Weinberg AC Thoreson GR Pierorazio P Matulay JT. Predicting renal parenchymal loss after nephron sparing surgery. J Urol. (2015) 194:658–63. 10.1016/j.juro.2015.03.098 25818030

[8] Laganosky DD Filson CP Master VA. Surgical margins in nephron-sparing surgery for renal cell carcinoma. Curr Urol Rep. (2017) 18:8. 10.1007/s11934-017-0651-5 28211006

[9] Cotta BH Meagher MF Bradshaw A Ryan ST Rivera-Sanfeliz G Derweesh IH. Percutaneous renal mass biopsy: historical perspective, current status, and future considerations. Expert Rev Anticancer Ther. (2019) 19:301–8. 10.1080/14737140.2019.1571915 30656989

[10] Richard PO Lavallée LT Pouliot F Komisarenko M Martin L Lattouf JB Finelli A. Is routine renal tumor biopsy associated with lower rates of benign histology following nephrectomy for small renal masses? J Urol. (2018) 200:731–6. 10.1016/j.juro.2018.04.015 29653161

[11] Berczi C Flasko T. Renal tumor in pregnancy: a case report and review of the literature. Urol Int. (2017) 99:367–9. 10.1159/000437337 26279416

[12] Sadat-Khonsari M Papayannis M Schriefer P Kluth L Meyer C Schüttfort V. Worth a second look: outcomes of patients with initial finding of regular renal tissue in CT-guided renal tumor biopsies. World J Urol. (2018) 36:789–92. 10.1007/s00345-017-2170-x 29372355

[13] Vaillancourt B Oligny L Déry J Franc-Guimond J Soglio DB. Ossifying renal tumor of infancy: report of a case with positive WT1 immunohistochemistry and high mitotic index and review of the literature. Pediatr Dev Pathol. (2017) 20:511–6. 10.1177/1093526617693105 29187024

[14] Hori J Kobayashi S Tamaki G Azumi M Kakizaki H. Diagnostic efficacy of percutaneous renal tumor biopsy - concomitant use of frozen section to accurately diagnose renal tumor with necrosis. Gan To Kagaku Ryoho. (2017) 44:771–4. 28912407

[15] Kutikov A Uzzo RG. The R.E.N.A.L. nephrometry score: a comprehensive standardized system for quantitating renal tumor size, location and depth. J Urol. (2009) 182:844–53. 10.1016/j.juro.2009.05.035 19616235

[16] Northrup BE Jokerst CE Grubb RL III Menias CO Khanna G Siegel CL. Hereditary renal tumor syndromes: imaging findings and management strategies. AJR Am J Roentgenol. (2012) 199:1294–304. 10.2214/AJR.12.9079 23169721

[17] Noventa A Herpe G Vesselle G Guibal A Velasco S Chan P. Chart for renal tumor microwave ablation from human study. Diagn Interv Imaging. (2018) 99:609–14. 10.1016/j.diii.2018.05.005 29914815

[18] Al-Lamki RS Mayadas TN. TNF receptors: signaling pathways and contribution to renal dysfunction. Kidney Int. (2015) 87:281–96. 10.1038/ki.2014.285 25140911

[19] Szkandera J Stotz M Absenger G Stojakovic T Samonigg H Kornprat P. Validation of C-reactive protein levels as a prognostic indicator for survival in a large cohort of pancreatic cancer patients. Br J Cancer. (2014) 110:183–8. 10.1038/bjc.2013.701 24201751 PMC3887299

[20] Perez-Ardavin J Sanchez-Gonzalez JV Martinez-Sarmiento M Monserrat-Monfort JJ García-Olaverri J Boronat-Tormo F. Surgical treatment of completely endophytic renal tumor: a systematic review. Curr Urol Rep. (2019) 20:3. 10.1007/s11934-019-0864-x 30649644

